# *Mycobacterium tuberculosis* infection in grazing cattle in central Ethiopia

**DOI:** 10.1016/j.tvjl.2010.05.005

**Published:** 2011-06

**Authors:** Gobena Ameni, Martin Vordermeier, Rebuma Firdessa, Abraham Aseffa, Glyn Hewinson, Stephen V. Gordon, Stefan Berg

**Affiliations:** a Aklilu Lemma Institute of Pathobiology, Addis Ababa University, PO Box 1176, Addis Ababa, Ethiopia; b TB Research Group, Veterinary Laboratories Agency, Weybridge, New Haw, Addlestone, Surrey KT15 3NB, UK; c Armauer Hansen Research Institute, PO Box 1005, Addis Ababa, Ethiopia; d College of Life Sciences, University College Dublin, Belfield, Dublin 4, Ireland; e UCD Conway Institute, University College Dublin, Belfield, Dublin 4, Ireland

**Keywords:** *Mycobacterium tuberculosis*, Tuberculosis, Cattle, Human, Transmission, Ethiopia

## Abstract

A preliminary study to characterise mycobacteria infecting tuberculous cattle from two different management systems in central Ethiopia was carried out. Approximately 27% of isolates from grazing cattle were *Mycobacterium tuberculosis*, while cattle in a more intensive-production system were exclusively infected with *M. bovis*. The practice of local farmers discharging chewed tobacco directly into the mouths of pastured cattle was identified as a potential route of human-to-cattle transmission of *M. tuberculosis*.

Mycobacteria of the *Mycobacterium tuberculosis* complex cause tuberculosis (TB) in various mammalian hosts but exhibit specific host tropisms (Smith et al., 2006). Bacterial species within the complex share 99.9% or greater similarity at the nucleotide level, and have a virtually identical 16S rDNA gene sequence (Sreevatsan et al., 1997; Brosch et al., 2002). The two major pathogenic species in this complex are *M. tuberculosis* and *M. bovis*, the causative agents of TB in humans and cattle, respectively. However, it is well known that *M. bovis* is zoonotic, while infection with *M. tuberculosis* has been sporadically reported in domestic and wild animal species, most frequently in animals living in prolonged, close contact with humans (Steele, 1980; Montali et al., 2001; Pavlik et al., 2003; Alfonso et al., 2004). Among domestic animals, infection with *M. tuberculosis* has been most frequently identified in cattle (Boulahbal et al., 1978; Sulieman, 2002; Prasad et al., 2005; Berg et al., 2009; Chen et al., 2009).

In Ethiopia, TB is prevalent in humans (0.6% prevalence; WHO, 2008) and livestock, as indicated by tuberculin test and slaughterhouse data (Ameni et al., 2007; Berg et al., 2009; Demelash et al., 2009). Previously, we found a disease prevalence of 10% in cattle from the Selalle region north of Addis Ababa and in animals in Holeta in the central highlands. These regions are major dairy farming areas that supply mainly unpasteurised milk to the urban and peri-urban areas of Addis Ababa (Ameni et al., 2007). In the present study, mycobacteria isolated from 52 cattle from these regions were characterised to the species level by molecular typing. Thirty of these animals were from the same intensive-production farm in Holeta and 22 were grazing cattle from 15 different farms in the Selalle region.

Heat-killed isolates were shipped frozen to the Veterinary Laboratories Agency (UK) for genotyping by multiplex PCR (Wilton and Cousins, 1992). Non-tuberculous mycobacteria were identified by GenoType Mycobacterium CM and AS kits (Hain Lifescience GmbH) or were sequenced at the 16S rDNA locus (Han et al., 2002) followed by sequence analysis (Berg et al., 2009). To discriminate isolates of the *M. tuberculosis* complex to the species level, ‘region of difference’ (RD) typing was carried out (Parsons et al., 2002) with forward, reverse, and internal primers for RD4 and RD10 (Brosch et al., 2002), and for RD9 (Berg et al., 2009). Spoligotyping of *M. tuberculosis* complex strains was also carried out (Kamerbeek et al., 1997).

The results of molecular typing of these isolates are summarised in Table 1. All 30 isolates from the intensive-production farm in Holeta were identified as *M. bovis*. The vast majority of cattle on this farm had tuberculous lesions in the lungs and thoracic lymph nodes. Of the 22 isolates from the grazing cattle in Selalle, one was identified as *M. bovis* and six as *M. tuberculosis.* These six isolates were isolated from six cattle obtained from six farms. Most of the *M. tuberculosis* isolates were recovered from lesions in the mesenteric and retropharyngeal lymph nodes. The *M. bovis* isolate had been cultured from a bronchial lymph node. Over 60% of the isolates from the grazing animals were non-tuberculous mycobacteria, as previously reported in Ethiopia (Berg et al., 2009).

Humans suffering from active TB are the most probable source of *M. tuberculosis* in animals, with infection spread via sputum, and rarely urine or faeces (Thoen and Steele, 1995). Reports of TB in cattle due to *M. tuberculosis* infection are usually from developing countries with a high prevalence of human TB. Infection rates of 6.2% and 7.4% have been reported in Algeria and Sudan, respectively (Boulahbal et al., 1978; Sulieman, 2002), and a recent slaughterhouse study from Ethiopia indicated that around 7% of isolates were *M. tuberculosis* (Berg et al., 2009). In the current study, approximately 27% of isolates from grazing cattle from Selalle were *M. tuberculosis.* This high incidence may not be representative of infection in the wider bovine population in Ethiopia, but does raise potentially serious zoonotic concerns.

In follow-up investigations we identified a unique habit among farmers in the Selalle region of chewing ground, baked tobacco and discharging the juice directly into the oral cavity of cattle (Fig. 1). This practice is considered a traditional anti-parasitic treatment that, is claimed, enhances animal performance. It is thus possible that such ‘mouth-to-mouth’ contact facilitates the transmission of *M. tuberculosis* from humans to cattle. Supportive evidence of this oral route of infection was the finding of tuberculous lesions in the retropharyngeal and mesenteric lymph nodes of such cattle post-mortem. In contrast, all *M. bovis* isolates from the intensive-production farm at Holeta were associated with respiratory tract lesions, supporting aerosol transmission between animals as the most likely route of infection (Ameni et al., 2006).

However, it is possible that the *M. tuberculosis*-infected cattle became infected through other means. For example, in Ethiopia, grazing cattle are commonly brought into the farmers’ households at night where they may become infected via aerosol transmission from humans. However, it would be anticipated that this would result in a greater degree of respiratory tract disease in the animals. Although no isolates of *M. tuberculosis* were collected from humans on the farms under study, spoligotyping identified the six *M. tuberculosis* isolates from the cattle as SIT149, a type previously found in humans in central Ethiopia (Brudey et al., 2006; G. Ameni, personal communication). However, direct epidemiological links between farmers and cattle infected with *M. tuberculosis* remain to be elucidated.

In conclusion, this study highlights the possible risk of human-to-cattle transmission of *M. tuberculosis* through the practice of mouth-to-mouth feeding of tobacco juice and/or where animals live in close contact with tuberculous humans. Epidemiological studies are ongoing to determine the impact of tobacco juice feeding on cattle health and on the potential for transmitting *M. tuberculosis* to cattle.

## Conflict of interest statement

None of the authors of this paper has a financial or personal relationship with other people or organisations that could inappropriately influence or bias the content of the paper.

## Figures and Tables

**Fig. 1:**
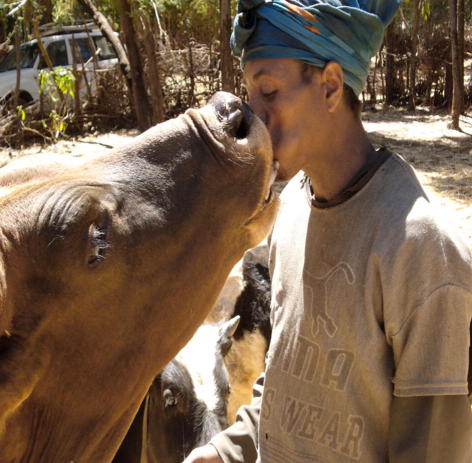
A farmer in central Ethiopia discharging tobacco juice directly into the oral cavity of his cattle, a common practice in this region and a possible route of transmission of *Mycobacterium tuberculosis* from humans to cattle.

**Table 1:** Details of mycobacterial isolates, farms types and tissues infected in cattle from central Ethiopia.

Isolate	Farm type	Tissue infected	Number of isolates
*Mycobacterium bovis*	Intensive	Lung, thoracic LN	30
*Mycobacterium bovis*	Grazing	Bronchial LN	1
*Mycobacterium tuberculosis*	Grazing	Bronchial,^a^ mesenteric and retropharyngeal LN	6
*Mycobacterium avium* sub-species	Grazing	Medial retropharyngeal, mesenteric, hepatic and cranial mediastinal LN, lung	6
*Mycobacterium gordonae*	Grazing	Caudal mediastinal LN	1
*Mycobacterium arupense*	Grazing	Left bronchial LN	1
*Mycobacterium vaccae*	Grazing	Mesenteric and medial retropharyngeal LN	2
*Mycobacterium holsaticum*	Grazing	Caudal mediastinal LN	1
Other Mycobacteria	Grazing	Medial retropharyngeal, mesenteric and caudal mediastinal LN	4

Total			52

LN, lymph node.

a One isolate.
